# Cellobiose phosphorylase from *Caldicellulosiruptor bescii* catalyzes reversible phosphorolysis via different kinetic mechanisms

**DOI:** 10.1038/s41598-022-08036-z

**Published:** 2022-03-10

**Authors:** Shaowei Bai, Liangzhen Yang, Honglei Wang, Chao Yang, Xuechen Hou, Jingjie Gao, Zuoming Zhang

**Affiliations:** 1 Key Laboratory for Molecular Enzymology and Engineering of the Ministry of Education, School of Life Science, Jilin University, Changchun, 130012 China; 2 School of Chemistry and Life Science, Changchun University of Technology, Changchun, 130012 China

**Keywords:** Enzymes, Molecular biology

## Abstract

In the process of yielding biofuels from cellulose degradation, traditional enzymatic hydrolysis, such as β-glucosidase catalyzing cellobiose, can barely resolve the contradiction between cellulose degradation and bioenergy conservation. However, it has been shown that cellobiose phosphorylase provides energetic advantages for cellobiose degradation through a phosphorolytic pathway, which has attracted wide attention. Here, the cellobiose phosphorylase gene from *Caldicellulosiruptor bescii* (*Cb*CBP) was cloned, expressed, and purified. Analysis of the enzymatic properties and kinetic mechanisms indicated that *Cb*CBP catalyzed reversible phosphorolysis and had good thermal stability and broad substrate selectivity. In addition, the phosphorolytic reaction of cellobiose by *Cb*CBP proceeded via an ordered Bi Bi mechanism, while the synthetic reaction proceeded via a ping pong Bi Bi mechanism. The present study lays the foundation for optimizing the degradation of cellulose and the synthesis of functional oligosaccharides.

## Introduction

Cellulose, the most abundant natural polymer on earth, is a sustainable green resource that is renewable, degradable, and biocompatible^1,2^. Due to its wide availability, large quantity and low acquisition cost, cellulose can be used to solve energy scarcity and resource regeneration problems^3^. Cellulose is a linear glucan composed of cellobiose, with glucose residues linked by β-1,4 glycosidic bonds^4^. Due to the stable molecular structure, cellulose needs to be broken down into monosaccharides or polysaccharides to convert into sustainable biofuels and other value-added products^5,6^. However, in addition to a mass of cellulose, plant cell wall also includes hemicellulose and lignin^7^. If large-scale degradation of cellulose is required, it needs to be pretreated, which is a very complex and uneconomical process. Traditionally, dilute or concentrated sulfuric acid was used to pretreat cellulose^8^ In the pretreatment process, degradation time, temperature, and pH of system need to be optimized, and the liquid fractions and solid residues need to be separated efficiently^9,10^, which is a time-consuming, costly and environmentally unfriendly. Consequently, biodegradation, the enzymatic degradation of cellulose, a green, efficient, and promising strategy, was developed^11^.

Cellulases, also known as cellulase systems, are enzymes involved in cellulose degradation. Cellulases generally include the following three main types: endoglucanase, exoglucanase and β-glucosidases^12^. The endoglucanase acts on the non-crystalline region of the cellulose chain, resulting in new chain ends and oligosaccharides of different lengths^13^. The exoglucanase degrades the reducing or non-reducing ends of the cellulose chain, producing mainly cellobiose^14^. Meanwhile, β-glucosidase acts only on the non-reducing ends, hydrolyzing cellobiose and cello-oligosaccharides to glucose^15^. Thus, cellobiose is an important intermediate in the cellulose degradation process.

Cellobiose, the repeating unit of cellulose, is a β-1,4-linked disaccharide of glucose. The accumulation of cellobiose severely inhibits the activity of exoglucanase in cellulose degradation. Traditionally, β-glucosidase was used to hydrolyze cellobiose to avoid this inhibition^16^. β-glucosidase cleaves cellobiose into two glucose molecules in the presence of water, followed by the metabolism of glucose molecules through the glycolytic pathway to produce ethanol^17^. This method consumes two ATP molecules for the hydrolysis of one cellobiose molecule. From the perspective of bioenergy, the degradation of intracellular cellulose requires high levels of ATP hydrolysis to provide energy^18^. Given the importance of bioenergy storage, it is imperative to explore a high-performance pathway that consumes less ATP than the cellulose hydrolysis pathway with two ATP molecules.

Researchers have shown that the phosphorolytic pathway involving cellobiose phosphorylase is another potentially better pathway for large-scale biofuels production^19^. The pathway requires less ATP for cellobiose degradation than the pathway involving β-glucosidase. Additionally, functional oligosaccharides are low-molecular-weight carbohydrates that usually contain less than 20 monosaccharide units linked by glycosidic bonds, and have been widely used in various fields such as medicine, food, feed, and agriculture^20,21^. Previous studies have demonstrated that cellobiose phosphorylase belongs to the glycoside hydrolase 94 family, catalyzing the reversible phosphorolysis reaction, including phosphorolytic reactions (i. e. , forward reactions in which cellobiose and phosphoric acid are used as substrates to produce α-glucose (Glu) and α-glucose-1-phosphate (Glu-1P) in the presence of cellobiose phosphorylase) and synthetic reactions (i. e. , reverse reactions in which cellobiose and phosphoric acid are produced with Glu-1P and Glu as substrates in the presence of cellobiose phosphorylase). The Glu-1P produced in phosphorolytic reactions can then be converted to α-glucose-6-phosphate (Glu-6P) by phosphoglucomutase without the ATP. Subsequently, Glu-6P can enter both glycolysis and pentose phosphate pathways^22,23^. Thus, the reverse phosphorolysis catalyzed by cellobiose phosphorylase efficiently degrades cellulose and synthesizes of a wide range of functional oligosaccharides^24^. Nevertheless, the current cellobiose phosphorylases have many defects, such as short half-life and glucose inhibition in the synthetic reaction. The kinetic mechanisms may affect catalytic efficiency of enzyme, and previous studies mostly focused on the kinetic mechanism of phosphorolytic reaction catalyzed by cellobiose phosphorylase, however, there are few studies on the kinetic mechanism of synthetic reaction by cellobiose phosphorylase. Therefore, it is important to find novel cellobiose phosphorylases with excellent enzymatic properties and to explore the kinetic mechanisms of synthetic reactions.

**Figure 1:**
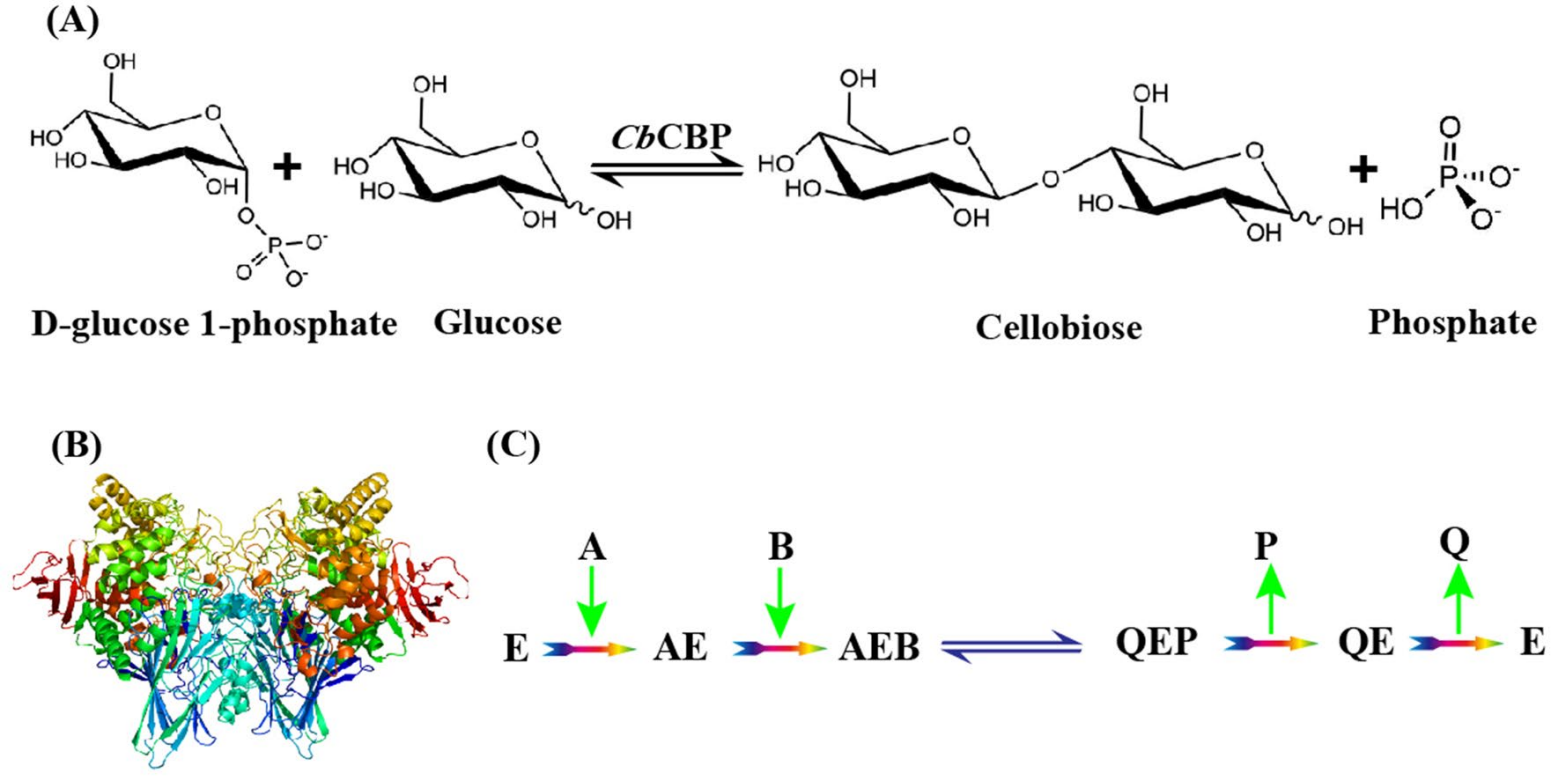
(**A**) Reversible phosphorolysis reaction catalyzed by *Cb*CBP. (**B**) Three-dimensional structure of the *Cb*CBP (PDB-entry 3qde) through homology modeling. The structure of the *Cb*CBP monomer consists of four distinct regions: an N-terminal domain, a helical linker region, an α-helix barrel domain, and a C-terminal domain. (**C**) Substrate binding and product release order for phosphorolytic reactions (E, *Cb*CBP; A, Pi; B, cellobiose; P, Glucose; Q, G-1-P).

*Caldicellulosiruptor bescii* is a thermophilic strain capable of expressing abundant cellulase. The whole-genome sequence of this strain revealed two potential cellobiose phosphorylase genes in its genome^25^. The present study investigated one of the cellobiose phosphorylase genes (GenBank ID: ACM59592.1), *Cb*CBP. The enzymatic characteristics and kinetic mechanism of the reversible phosphorolysis reaction catalyzed by *Cb*CBP were investigated (Fig. 1A). The results showed that *Cb*CBP had good thermal stability, high glucose tolerance, and broad substrate selectivity. Besides, the reaction kinetics indicated that the synthetic reaction and phosphorolytic reaction by *Cb*CBP presented different kinetic mechanisms in vitro.

## Results

### Cloning and purification of *Cb*CBP from *C. bescii*

The *Cb*CBP gene was cloned from the genomic DNA of *C. bescii* DSM 6725. A homology modeling conducted by the SWISS-MODEL (https://swissmodel.expasy.org/) to understand the structure of the *Cb*CBP. In our homology modeling, three protein structures (PDB ID 3qde, 3rsy and 3s4c) from the Protein Data Bank (https://www.rcsb.org/) were chosen as alternative templates. The alignment of the target and templates protein sequences indicates the sequence identity of 73% (for PDB ID 3qde), 60.07% (for PDB ID 3rsy) and 59.95% (for PDB ID 3s4c) respectively (Fig S1). The evaluation of models quality was listed in Table S2, in which 98.3% residues of the model (built by PDB ID 3qde), 97.5% residues of the model (built by PDB ID 3rsy) and 97.5% residues of the model (built by PDB ID 3s4c) are in the allowed region using the PROCHECK method for assessing the "stereochemical quality" of alternative templates^26^. The MolProbity is a web server offering quality validation for 3D structures of proteins, in which the model (built by PDB ID 3qde) shows the highest percentage (95.3%) in the favored regions compared with the other templates^27^. The ERRAT method is a protein structure based verification algorithm, used to analyze the statistics of non-bonded interactions between different atom types in a target protein structure compared with statistics from highly refined structures^28^. The result in the ERRAT section of the Table S2 shows that the ERRAT score for the three models are 95.301, 92.327 and 93.013 respectively, indicating that the three models are reliable. VERIFY3D determines the compatibility of an atomic model (3D) with its own protein sequence (1D) by assigning a structural domain and comparing the results to good structures^29,30^. The VERIFY3D score (95.93% of the residues had an averaged 3D-1D score > 0.2) of the model (built by PDB ID 3dqe) is higher than the other models (built by PDB ID 3rsy and 3s4c), showing the most reliable modeling structure. The above results proved that our homology modeling was reliable and the optimal *Cb*CBP structure template was PDB ID 3dqe. Analysis of the amino acid sequence of the template protein (PDB ID 3qde) revealed that two identical subunits (chain A and chain B) formed dimers, which were linked by a series of hydrophobic interactions and hydrogen bonds. From the results of homology modeling, we concluded that *Cb*CBP was also present as a homo-dimer^31^, and its secondary structure consisted of four domains: an N-terminal domain, a helical linker region, an α-helix barrel domain, and a C-terminal domain (PDB ID 3qde) (Fig. 1B)^32^.

**Figure 2:**
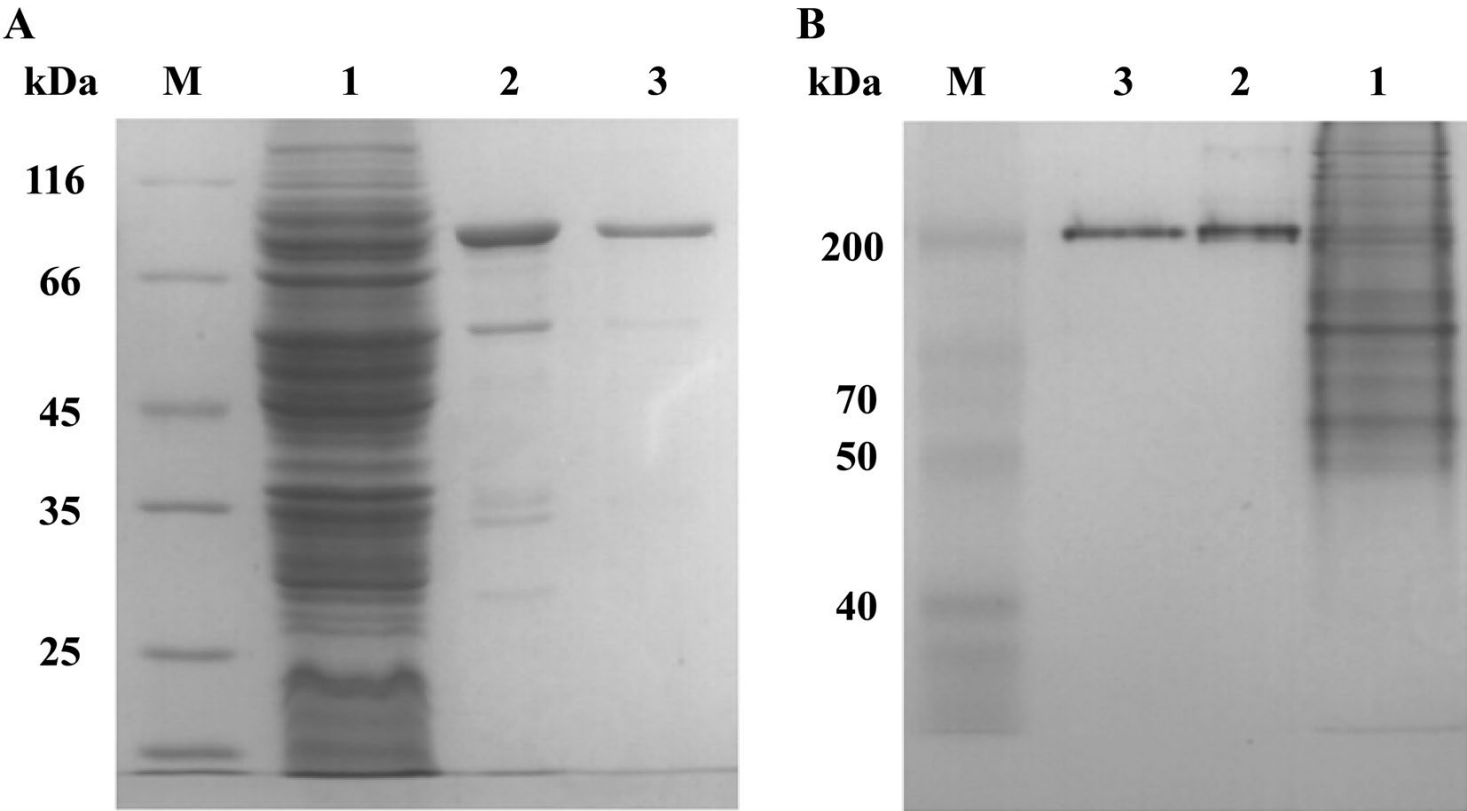
SDS-PAGE (**A**) and native-PAGE (**B**) of *Cb*CBP at different purification steps. Lane M, molecular mass markers; lane 1, thermal treatment; lane 2, Ni–NTA fraction; lane 3, Q sepharose fraction. (The original gels were presented in Fig S2.).

To further determine the characteristics and kinetic mechanism of the *Cb*CBP, the gene was cloned into the plasmid pET20b and expressed in *E. coli* BL21 (DE3) cells. The recombinant *Cb*CBP was purified to homogeneity by thermal treatment, Ni–NTA agarose column purification and Q sepharose chromatography. Purified *Cb*CBP showed a single band of 94 kDa on SDS-PAGE (Fig. 2A), consistent with the theoretical molecular mass. The molecular mass on native-PAGE was about 200 kDa (Fig. 2B), indicating *Cb*CBP as a dimeric protein, consistent with the results of homology modeling and the previously reported cellobiose phosphorylases such as *Ct*CBP^32^, *Tm*CBP^33^, and *Ra*CBP^22^. Meanwhile, few cellobiose phosphorylases, such as CepA^34^ and *Cu*CBP^35^, exist as monomers.

### Effect of pH and temperature on enzyme activity and stability of *Cb*CBP

**Figure 3:**
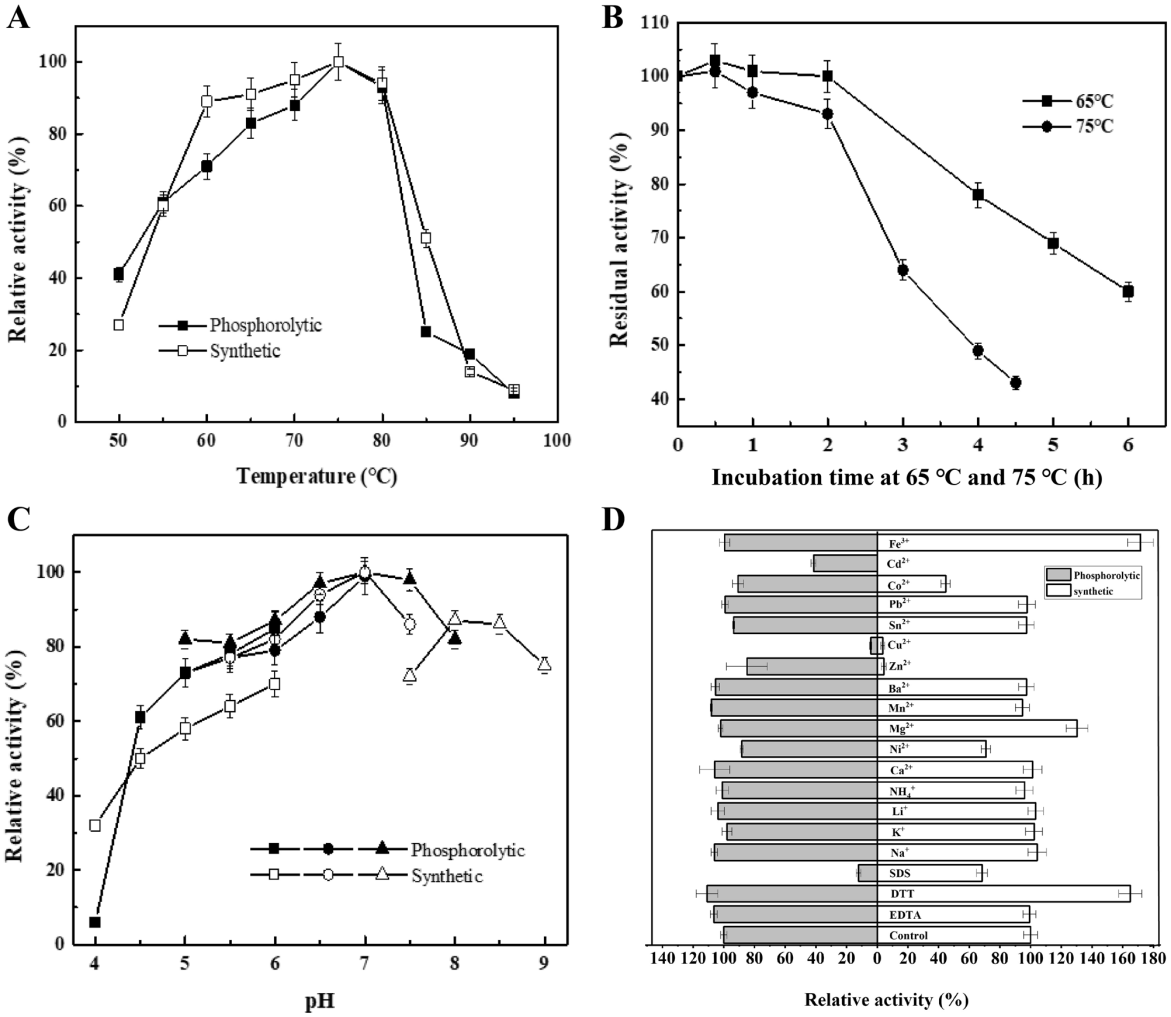
Characterization of *Cb*CBP enzymatic properties. (**A**) Optimum temperature. the enzyme activity was measured at various temperatures (50–95 °C) for 30 min in 20 mM MES buffer (pH 7.0). Values are shown as a percentage of the maximum activity, defined as 100%. (**B**) Thermal stability of *Cb*CBP. The enzyme was incubated at 65 °C (filled square) and 75 °C (filled Circle) in 20 mM MES buffer for different durations. Residual activity was measured following the standard method. (**C**) Optimum pH. The enzyme activity was measured at 75 °C for 30 min in 50 mM phosphorolytic reaction buffer at various pH levels: sodium citrate (filled square), MES (filled Circle), PB (filled triangle), synthetic reaction buffer: sodium citrate (open square), MES (open circle), Tris (open triangle). Values are shown as a percentage of the maximum activity, defined as 100%. (**D**) Effects of various metal ions and some organic reagents. (The data of all experiments are presented as the means of triplicate experiments with standard deviations, no repeated comments will be made below.).

Enzyme activity is essential for the application of enzyme molecules in various fields. The present study determined the optimum temperature, optimum pH, temperature stability, and metal ions’ effect on the activity of the *Cb*CBP. As shown in Fig. 3A, the optimum temperature for *Cb*CBP in phosphorolytic and synthetic reactions was 75 °C. The enzyme activity decreased sharply with increasing temperature after 80 °C. The enzyme activity in the synthetic reaction was significantly higher than that in the phosphorolytic reaction at the same temperature. The temperature stability of *Cb*CBP was further examined, and the results are displayed in Fig. 3B. The half-life of the enzyme was 6 h and 4 h at 65 °C and 75 °C, respectively. Moreover, the residual activity of *Cb*CBP remained above 90% after incubation at 65 °C and 75 °C for 2 h. Compared to *Ra*CBP^22^, which can maintain more than 90% enzyme activity only below 40 °C, *Cb*CBP exhibited higher temperature stability. Then, the optimum pH of the enzyme was determined. As shown in Fig. 3C, the optimum pH for both synthetic reaction and phosphorolytic reaction were 7.0. The relative enzyme activity in the synthetic reaction at pH 4.0 was 32% of the maximum enzyme activity, which was five times higher than that of phosphorolytic reaction (6%). Compared with *Ct*CBP^36^, the relative enzyme activity of *Cb*CBP remained at about 50% at pH 4.5, while *Ct*CBP was only about 30%. Similarly, *Cb*CBP maintained about 80% of its enzyme activity at pH 8, while *Ct*CBP maintained about 60%. These results suggested that *Cb*CBP had good pH stability compared to cellobiose phosphorylase from other sources. Meanwhile, the relative enzyme activity of the synthetic reaction at pH 9.0 was 75%, which indicated that the pH tolerance range of *Cb*CBP in the synthetic reaction was broader than that in phosphorolytic reaction. Finally, the effects of metal ions and chemical reagents on the activities of *Cb*CBP in phosphorolytic and synthetic reactions were determined (Fig. 3D). Monovalent metal ions had little effect on *Cb*CBP enzymatic activity. In contrast, divalent metal ions (Cu^2+^, Cd^2+^, Ni^2+^, and Zn^2+^) and organic reagents (SDS) inhibited phosphorolytic and synthesis reactions to varying degrees, while Mg^2+^, Fe^3+^ and DTT boosted the activity. The effect of metal ions on enzyme activity is dissimilar in different enzymes. We speculated that it may be related to the properties of enzyme molecules themselves, and the specific molecular mechanism needs to be further studied. However, it can be predicted that the different effects of metal ions on enzyme activity would affect the practical application of enzymes. The inhibition of enzyme activity by SDS and the promotion of enzyme activity by DTT were also found in keratinase^37^. These results indicated the excellent thermal stability and acid–base resistance of the enzyme, which is desirable for application in various fields.

### Substrate selectivity for the phosphorolytic and synthetic reactions

The phosphorolytic activity of *Cb*CBP on different disaccharides (cellobiose, cellotriose, cellotetrose, maltose, trehalose, lactose, and sucrose) was determined in the presence of Pi. *Cb*CBP showed high activity on cellobiose but did not phosphorylate cellooligosaccharides, such as cellotriose and cellotetrose. The specific activity on cellobiose was 2 U/mg. Besides, *Cb*CBP could not cleave cellobiose in the absence of inorganic phosphate. Therefore, it is presumed that *Cb*CBP cleaved the β-1,4 glucosidic bond specifically and recognized disaccharides but had no effect on trisaccharides and tetrasaccharides. The synthetic activity was examined in the presence of 10 mM Glu-1P using various monosaccharides and disaccharides as glucosyl acceptors. *Cb*CBP utilized D-xylose, D-glucose, D-mannose, D-arabinose and D-fructose at a rate in decreasing order (Table 1). These results suggest that *Cb*CBP can catalyze the synthesis of the corresponding disaccharide using the above monosaccharides as acceptors but not polysaccharides using disaccharide as an acceptor. Interestingly, D-xylose was more suitable than D-glucose as optimal substrate for *Cb*CBP in synthetic reactions compared with those in which *Ra*CBP^22^, *Ct*CBP^38^ and *Cg*CBP^39^ had the highest activity towards D-glucose. However, the reason for our observed substrate selectivity needs to be elucidated by further experiments and molecular dynamics.

**Table 1:** Substrate selectivity of *Cb*CBP in the phosphorolytic and synthetic reactions.

Reaction	Substrate	Rate (U/mg)
Phosphorolysis^a^	Cellobiose	2.07 ± 0.10
Synthese^b^	D-xylose	3.58 ± 0.12
	D-glucose	3.28 ± 0.54
	D-mannose	0.70 ± 0.01
	D-Arabinose	0.35 ± 0.01
	D-Fructose	0.21 ± 0.01

^a^The following substrates were less than 10% of relative activity in phosphorolytic reactions: maltose, cellotriose, cellotetrose, and trehalose (detailed data not listed).

^b^The following acceptors were less than 5% of relative activity in synthetic reactions: D-sorbitol, D-mannitol, methyl-α-D-glucoside, L-arabinose, D-galactose, D-ribose, lactose, cellobiose, maltose, cellotriose, trehalose, and sucrose (detailed data not listed).

### Kinetic mechanism of the phosphorolytic reaction

**Figure 4:**
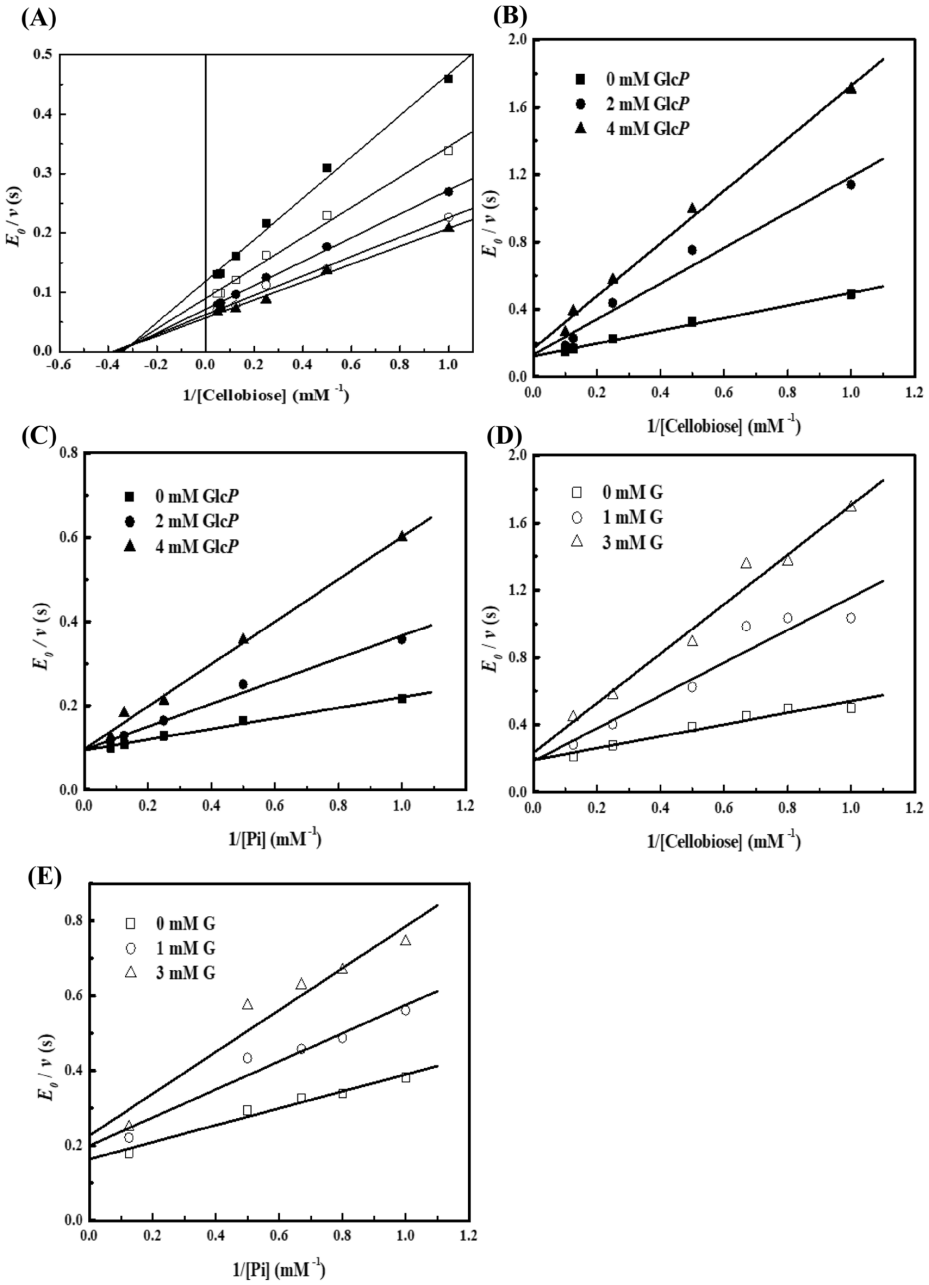
Kinetic mechanisms of the phosphorolytic reaction. (**A**) Double reciprocal plots of cellobiose phosphorolysis catalyzed by *Cb*CBP. The concentration of Pi was as follows: 1 mM (filled square), 2 mM (open square), 4 mM (filled Circle), 8 mM (open circle), and 12 mM (Filled triangle). R^2^ > 0.99. (**B**–**E**) Inhibition patterns of the products against the substrates. The other substrates were 1 mM Pi (B, D) and 2 mM cellobiose (C, E). B: Glu-1P against cellobiose, C: Glu-1P against Pi, D: glucose against cellobiose, E: glucose against Pi. filled square and open square: none of the initial product, filled Circle: 2 mM Glu-1P, fillied triangle: 4 mM Glu-1P, open circle: 1 mM glucose, open triangle: 3 mM glucose.

The initial reaction rates of *Cb*CBP at different concentrations of cellobiose (1–20 mM) and Pi (0.5–12 mM) were measured under the standard conditions at 70 °C and pH 7.0 to explore the kinetic mechanism of the phosphorolytic reaction catalyzed by *Cb*CBP. The kinetic analysis diagram was plotted using the inverse of the cellobiose concentration (1/[Cellobiose]) as the horizontal coordinate and the inverse of the reaction catalytic constant (E_0_/*v*) as the vertical coordinate (Fig. 4A). Double reciprocal plots of the initial velocities against various initial cellobiose concentrations and five fixed Pi concentrations gave a series of lines intersecting at the upper left-hand quadrant. These observations indicated that the phosphorolytic reaction of *Cb*CBP followed an ordered Bi Bi mechanism. The kinetic parameters in theoretical Eq. (1) were shown in Table 2.

**Table 2:** Kinetic parameters for the reaction of *Cb*CBP with various substrate.

Reaction	Km^A^ (mM)	Km^B^ (mM)	*K*cat (s^-1^)	Ki^A^ (mM)
Phosphorolysis^a^	2.8	1.2	18.4	3.3
Synthese^b^ (A)	35	116.3	294	no^c^

^a^A represents cellobiose, and B represents inorganic phosphorus.

^b^A represents D-glucose 1-phosphate, and B represents D-glucose.

^c^Ki^A^ value could not be calculated because there was no inhibition when the concentration of glucose reached 100 mM in the synthetic reaction.

The inhibition pattern of the products, Glu-1P and D-glucose, against the substrates, cellobiose and Pi, were further investigated. The results showed that Glu-1P acted as a mixed-type inhibitor against cellobiose (Fig. 4B) and a competitive inhibitor against Pi (Fig. 4C). In contrast, D-glucose acted as a mixed-type inhibitor of both cellobiose and Pi (Figs. 4D and 4E). These observations suggested that the phosphorolytic reaction catalyzed by *Cb*CBP is an ordered Bi Bi mechanism, in which Pi binds to *Cb*CBP before cellobiose, and Glu-1P is released after D-glucose. The specific sequence of substrate binding and product release of phosphorolytic reaction is shown in Fig. 1C. The substrate-binding order was the same as that of *Ct*CBP^38^. While *Cg*CBP^40^ and *Cu*CBP^35^ catalyzed the phosphorolytic reaction via the ordered Bi Bi mechanism, the substrate-binding order was reversed. Unlike these enzymes, several studies have shown that *Tm*CBP^33^ and *Ra*CBP^22^ catalyzed the phosphorolytic reactions via random-ordered Bi Bi mechanisms. In brief, the binding of the substrates, cellobiose and Pi, was random, while the products release was ordered.

### Kinetic mechanism of the synthetic reaction

**Figure 5:**
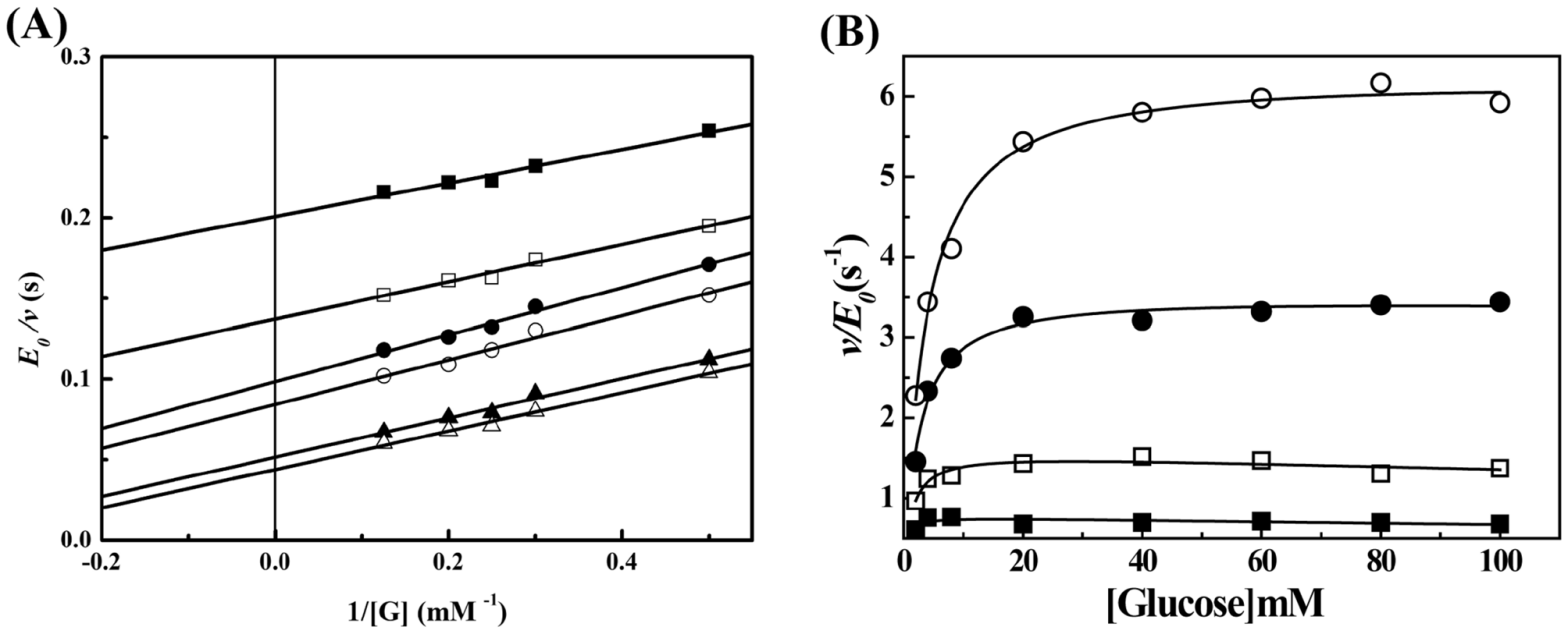
Kinetic mechanisms of the synthetic reaction. (**A**) Double reciprocal plots of the synthetic reaction catalyzed by *Cb*CBP. The concentrations of Glu-1P were as follows: 2 mM (filled square), 3 mM (open square), 4 mM (filled circle), 5 mM (open circle), 8 mM (Filled triangle) and 12 mM (open triangle). 0.98 < R^2^ < 0.99. (**B**) v-[D-glucose] Plot of the synthetic reaction of *Cb*CBP at fixed concentrations of Glu-1P. Glu-1P was used at 1 mM (filled square), 2.5 mM (open square), 5 mM (filled circle) and 10 mM (open circle) concentrations. The theoretical lines were obtained by regressing the experimental data to the Michaelis Menten equation.

The inverse of the substrate D-glucose concentration (1/[G]) was used as the horizontal coordinate, and the inverse of the reaction catalytic constant (E_0_/v) was used as the vertical coordinate to generate a double reciprocal plot to investigate the kinetic mechanism of *Cb*CBP in the synthetic reaction. As shown in Fig. 5A, the double reciprocal plots gave a series of lines more parallel than intersecting, this parallel line pattern is consistent with the ping pong kinetics for the phosphorolysis of wild-type sucrose phosphorylase from *Leuconostoc mesenteroides*^41^. Based on these data, we inferred that the synthetic reaction of *Cb*CBP may follow a ping pong Bi Bi mechanism rather than an ordered Bi Bi mechanism. To further confirm our speculation, we investigated whether the Lineweaver–Burk plot of the ternary complexes formed in the ordered Bi Bi mechanism also fit the parallel line pattern. In contrast to previous literature, however, the double reciprocal plot of ternary complex has not been found to fit the parallel line pattern, such as uridine phosphorylase (*Trypanosoma cruzi*) and 4-O-β-D-mannosyl-D-glucose phosphorylase (*Rhodothermus marinus*), and trehalose 6-phosphate phosphorylase (*Lactococcus lactis* ssp. *lactis*)^42–44^. In other words, no data was found on the association between ternary complex and ping pong mechanism. Therefore, it could be tentatively determined that the synthetic reaction of *Cb*CBP formed a ping pong kinetic mechanism instead of forming a ternary complex. In general, in addition to the initial-velocity patterns, the presence or absence of exchange reactions and other stereochemical evidence can also be used to determine whether the enzyme catalyzes reactions by a ping pong mechanism^45,46^, which will be done in further research in the future.

For synthetic reactions catalyzed by *Cb*CBP, the kinetic parameters in theoretical Eq. (2) were shown in Table 2. Unlike the synthetic reactions catalyzed by *Cg*CBP^39^ and *Tm*CBP^33^ from other sources, which catalyzed synthetic reactions via an ordered Bi Bi mechanism, the synthetic reaction catalyzed by the *Cb*CBP followed a ping pong Bi Bi mechanism and differed from the phosphorolytic reactions. We speculated that these differences in the kinetic mechanism might improve the catalytic efficiency of the enzyme. Previous studies have shown that the low concentration of the substrate D-glucose remarkably inhibited the synthetic reaction catalyzed by cellobiose phosphorylase, which might affect the synthesis efficiency of functional oligosaccharides. For example, D-glucose at a low concentration (≤ 10 mM) in the synthetic reaction competitively inhibited *Cg*CBP^39^ and *Ct*CBP^38^. Meanwhile, *Ra*CBP can tolerate 20 mM D-glucose in a competitive-uncompetitive manner^22^. Here, the initial velocities of *Cb*CBP for D-glucose (1–100 mM) and Glu-1P (1–10 mM) at different concentrations were also investigated. As shown in Fig. 5B, typical hyperbolic curves were obtained, and the reaction appeared to proceed following the Michaelis–Menten equation. Notably, the initial velocity of the synthetic reaction catalyzed by *Cb*CBP did not decrease, even at a D-glucose concentration of 100 mM, suggesting tolerance of *Cb*CBP to D-glucose inhibition. This phenomenon may be due to the different catalytic mechanisms of the synthetic reactions by *Cb*CBP with the cellobiose phosphorylase mentioned above. This result has further strengthened our conviction that synthetic reaction of *Cb*CBP possesses an especial kinetic mechanism distinct from other cellobiose phosphorylases, namely ping pong Bi Bi mechanism. In general, the excellent glucose tolerance of *Cb*CBP provides a possibility for the efficient synthesis of functional oligosaccharides.

## Conclusions

The present study generated a cellobiose phosphorylase from the genome of *C. bescii*. The *Cb*CBP was cloned, expressed, and purified and then assayed in vitro for degrading cellobiose and synthesizing oligosaccharides. The analysis of the enzymatic properties and kinetic mechanisms revealed that the half-life of the *Cb*CBP was 6 h and 4 h at 65 °C and 75 °C, indicating that good thermal stability. Additionally, the temperature and pH tolerance range of the *Cb*CBP-catalyzed synthetic reaction were wider than that of the phosphorolytic reaction. Intriguingly, the *Cb*CBP is the first unique cellobiose phosphorylase found to catalyze synthetic and phosphorolytic reactions via different kinetic mechanisms. Compared with cellobiose phosphorylases from other sources, *Cb*CBP exhibited high tolerance to D-glucose concentration, which may be due to the ping pong Bi Bi mechanism of its synthetic reaction. However, the elaborate mechanisms need to be elucidated in further research, in which the stereochemical study would be used as a means of determining the kinetic mechanisms of *Cb*CBP. In summary, the distinct properties of *Cb*CBP would facilitate its application in the biodegradation of cellulose and the synthesis of functional oligosaccharides.

## Methods

### Materials

Cellobiose, D-glucos, D-xylose, methyl-α-D-glucoside, methyl-β-D-glucoside, D-mannitol, D-glucitol, *N*-acetyl-D-glucosamine, D-glucose-1-phosphate, L-arabinose, D-frutose, D-galactose, lactose, laminaribiose, and glucose 1-phosphate (Glu-1P) were purchased from Sigma-Aldrich (USA).

### Cloning and sequencing of *Cb*CBP and construction of expression plasmid

The *Cb*CBP gene sequence (GenBank ID: ACM59592.1) was obtained from Cazy (http://www.cazy.org/) by overlap PCR using genomic DNA of *Caldicellulosiruptor bescii* DSM 6725 prepared by the CTAB method^47^. Corresponding two pairs of primers were designed with Oligo software (http://www.oligo.net/) (Table S1), and *Nco*I and *Xho*I were used as the restriction enzyme cutting sites. PrimeSTAR HS DNA polymerase (TaKaRa Bio, Dalian, China) was used for PCR. The PCR products were ligated into the pET 20b ( +) vector (Novagen, Madison, WI, USA) with the same restriction sites using T4 DNA ligase (NEB). The ligation product (10 µL) was transformed into competent *E. coli* XL1-Blue cells and inoculated on LB (Luria–Bertani) plate containing 100 µg/mL ampicillin. The positive clones were confirmed by sequencing. Then the recombinant *Cb*CBP with (His)_6_ residues at the C-terminal was transferred into *E. coli* BL21 Codonplus-RIL cells.

### Expression and purification of cellobiose phosphorylase

The transformed cells were cultured in auto-inducing media (1 L; Trypton 10 g, Yeast 5 g, 50*M 20 mL, 50*5052 20 mL, 1 M MgSO_4_ 2 mL) with 100 µg/mL ampicillin overnight at 37 °C in a rotary shaker (150 rpm) to produce the seed culture^48^. Then, 10 mL of the seed culture was used as the inoculum for 1000 mL of the auto-inducing media supplemented with 100 µg/mL ampicillin. After 4 h, cells were induced with lactose and cultured for 13 h at 30 °C (110 rpm). The cells were harvested by centrifugation at 8000 rpm for 30 min at 4 °C and suspended in 20 mM MES-NaOH buffer (pH 7.0) containing 500 mM NaCl, 30 mM imidazole (buffer A), and 1 mM phenylmethanesulfonyl fluoride (PMSF). The cells were fragmented by ultrasonication (Sonics VCX500, USA) at 4 °C. The ultrasonic sample was centrifuged, and the supernatant extracted was incubated at 70 °C for 20 min and centrifuged at 12,000 rpm for 30 min to remove the cell debris. The supernatant was filtered through a 0.22 µm microfiltration membrane to obtain the crude enzyme.

Subsequently, enzyme purification was carried out with an ÄKTA purifier (GE Healthcare) at room temperature and detected at an absorbance of 280 nm. The crude enzyme was applied to the HisTrap-FF column (1 mL, GE Healthcare), equilibrated with buffer A. The column was washed with buffer A until most of the nonspecific binding proteins were removed. The *Cb*CBP protein was eluted with 300 mM imidazole at a flow rate of 1 mL/min. The purified enzyme was dialyzed overnight at 4 °C against 20 mM Tris–HCl buffer (pH 7.5). Then, the dialyzed proteins were loaded onto the Sepharose high performance Q column (1 mL, GE Healthcare) equilibrated with 20 mM Tris–HCl buffer (pH 7.5). The enzyme was eluted using NaCl at a linear gradient from 0 to 1 M in Tris–HCl buffer (pH 7.5). The purified enzyme was dialyzed overnight at 4 °C against 20 mM MES-NaOH buffer (pH 7.0) and stored at − 20 °C. Finally, the purity of the enzyme obtained at each step was confirmed by sodium dodecyl sulfate–polyacrylamide gel electrophoresis (SDS-PAGE) and native polyacrylamide gel electrophoresis (native-PAGE).

### Analysis methods of *Cb*CBP, D-glucose, and Pi

The concentration of *Cb*CBP was measured using Easy Protein Quantitative Kit (Transgen Biotech) with bovine serum albumin (BSA) as a standard. D-glucose was measured by the glucose oxidase–peroxidase method using a Glucose Assay Kit (Huili Biotech. Ltd, Changchun, China). Pi was quantified following the procedure by Jeffrey D.G. and David R.B in the presence of Glu-1P^49^.

The standard condition for enzyme-catalyzed reaction is a modification of the previous method^36^. The enzyme reactions were carried out at 75 °C in 50 mM MES-NaOH buffer (pH 7.0). The initial rate of the phosphorolytic reaction was determined by measuring the amount of D-glucose during the reaction with 10 mM cellobiose and 10 mM Pi. The amount of Pi liberated from 10 mM α-Glu-1P was determined for the synthetic reaction.

### Effect of acid–base and temperature on enzyme activity and stability of *Cb*CBP

The effect of pH on the enzyme activity in the phosphorolytic and synthetic reactions was determined under the standard conditions instead of 50 mM MES-NaOH buffer (pH 7.0) by 50 mM various buffers: sodium citrate (pH 4.0–6.0), MES-NaOH (pH 5.0–7.0), and Tris–HCl (pH 7.5–9.0), The optimum pH for the phosphorolytic reaction of *Cb*CBP in buffers at pH ranging from 4.0 to 8.0 was determined at 75 °C using 10 mM cellobiose and 10 mM Pi as substrates. The optimum pH of *Cb*CBP synthetic reaction in buffers at pH ranging from 4.0 to 9.0 was determined at 75 °C using 10 mM D-glucose and 10 mM Glu-1P as substrates. Likewise, the optimum temperature was measured under standard conditions at various temperatures (50–95 °C). The thermal stability was evaluated by measuring the residual phosphorolytic activity under standard conditions after incubation of *Cb*CBP (0.14 mg/mL) at 65 °C and 75 °C for prolonged durations.

### Substrate selectivity for the phosphorolytic and synthetic reactions

The phosphorolytic activities of *Cb*CBP on different disaccharides (cellobiose, cellotriose, cellotetrose, maltose, trehalose, lactose, and sucrose) were examined under the standard conditions described above. Further, the synthetic reactions were performed by substituting D-glucose with various carbohydrate acceptor candidates (D-xylose, D-mannose, D-arabinose, D-fructose, D-sorbitol, D-mannitol, methyl-α-D-glucoside, L-arabinose, D-galactose, D-ribose, lactose, cellobiose, maltose, cellotriose, trehalose, and sucrose) under the standard conditions described above to investigate the acceptor specificities of *Cb*CBP.

### Analysis of the kinetic mechanism

The kinetic analysis of cellobiose phosphorolysis was carried out by measuring the initial velocities under the standard conditions with 230 nM of *Cb*CBP and different combinations of cellobiose (1–20 mM) and Pi (0.5–12 mM). The kinetic parameters of the phosphorolytic reaction were calculated by curve fitting the experimental data to the theoretical Eq. (1) for an ordered Bi Bi mechanism^38^

1 $$ \nu = {\text{ V}}_{{{\text{max}}}} \left[ {\text{A}} \right]\left[ {\text{B}} \right]/\left( {{\text{Ki}}^{{\text{A}}} {\text{Km}}^{{\text{B}}} + {\text{Km}}^{{\text{B}}} \left[ {\text{A}} \right] + {\text{Km}}^{{\text{A}}} \left[ {\text{B}} \right] + \left[ {\text{A}} \right]\left[ {\text{B}} \right]} \right) $$

where A represents cellobiose, and B represents inorganic phosphorus.

The order of substrate binding and product release was determined via a product inhibition analysis. D-glucose (0–3 mM) and Glu-1P (0–4 mM), against the substrates, cellobiose and Pi, were also measured at varying concentrations of cellobiose (1–10 mM) and 1 mM Pi, and varying concentrations of Pi (0.5–12 mM) and 4 mM cellobiose by measuring D-glucose and Pi, respectively.

The kinetic analysis of synthetic reactions was performed under the standard conditions with 430 nM *Cb*CBP and different combinations of D-glucose (2–8 mM) and Glu-1P (2–10 mM). The kinetic parameters were calculated by curve fitting the experimental data to the theoretical Eq. (2) for the ping pong mechanism^41^.

2 $$ \nu = {\text{ V}}_{{{\text{max}}}} \left[ {\text{A}} \right]\left[ {\text{B}} \right]/\left( {{\text{Km}}^{{\text{B}}} \left[ {\text{A}} \right] + {\text{Km}}^{{\text{A}}} \left[ {\text{B}} \right] + \left[ {\text{A}} \right]\left[ {\text{B}} \right]} \right) $$

where A represents Glu-1P, and B represents D-glucose.

### Analysis of D-glucose inhibition

In the synthetic reactions catalyzed by *Cb*CBP, D-glucose and Glu-1P were used as substrates, where the concentration of Glu-1P was a constant and the concentration of D-glucose was from 0 to 100 mM, the initial reaction rates were measured at different glucose concentrations. The corresponding curves were fitted by the Michaelis–Menten equation.

## Supplementary Information

Supplementary Information.
