# Supplementary material for: Cellobiose phosphorylase from Caldicellulosiruptor bescii catalyzes reversible phosphorolysis via different kinetic mechanisms
Source: Sci Rep. 2022 Mar 10;12:3978. doi: 10.1038/s41598-022-08036-z (PMC8913831; doi:10.1038/s41598-022-08036-z)
Supplement: Supplementary file 1 — Supplementary Information. [file 41598_2022_8036_MOESM1_ESM.pdf]

Supporting information

Cellobiose phosphorylase from *Caldicellulosiruptor bescii* catalyzes reversible phosphorolysis via different kinetic mechanisms

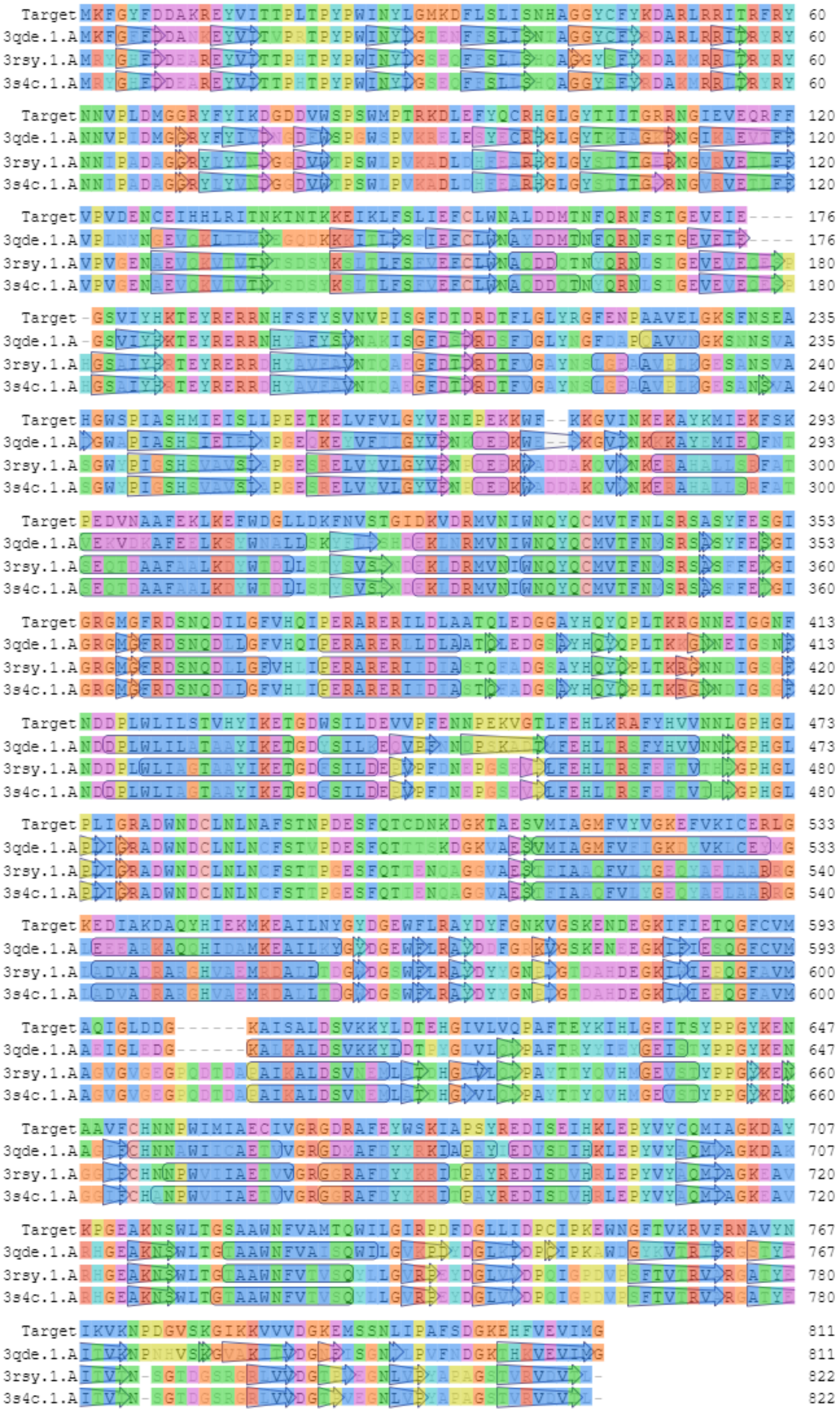

Figure. S1 Sequence alignment of homologous modeling (PDB ID 3qde, 3rsy, and 3s4c), the arrows represent the β-fold, the round rectangles represent the α-helix, and the rest represent the β-turn or random coil.

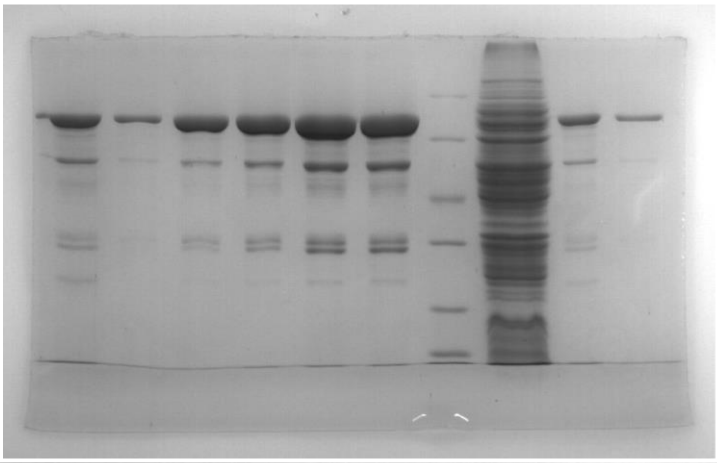

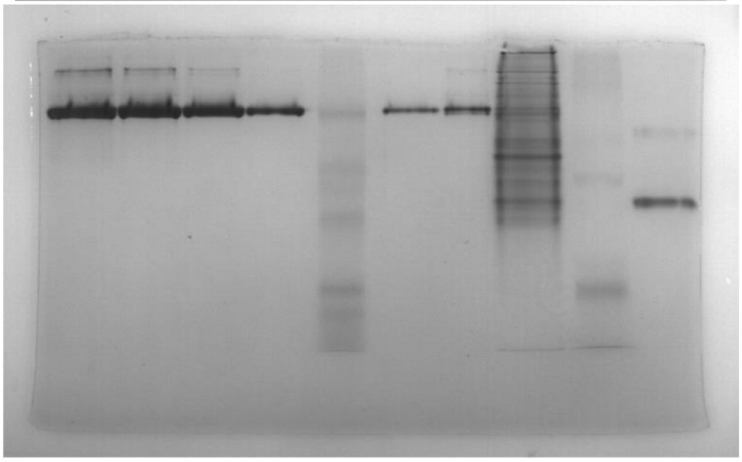

Figure. S2 The original gels of Figure 2 in the text (The top figure represents the original gel of SDS-PAGE and the bottom figure represents the original gel of native-PAGE)

Table S1 *Primers for constructions of expression plasmid.* In order to synthesize the target gene, F1 and R1 were used as upstream and downstream primers respectively, and genome was used as template to synthesize Fragment A. Then, F2 and R2 were used as upstream and downstream primers respectively, and genome was used as template to synthesize Fragment B. Finally, F1 and R2 were used as upstream and downstream primers respectively, and Fragments A and B were used as templates to synthesize target genes.

Gene	Primer name	Primer sequence (5'-3')
Fraction A	F1	AATCT<u>CCATGG</u>CTAAGTTTGGCTATTTTGACGATGCT
Fraction A	R1	AATCATAATCCACGGGTTGTTGTGATCACAACAACCCGTGGA
Fraction B	F2	TCACAACAACCCGTGGATTATGATT
Fraction B	R2	TACCGA<u>CTCGAG</u>TCCCATTATCACTTCAACAAAATGC

Table S2 *The evaluation of models quality using different score-based methods*

PDB ID	PROCHECK	MolProbity Ramachandran Favored region	MolProbity Ramachandran Allowed region	ERRAT	VERIFY3D
**3qde**	88% core 10.3% allow 0.5% gener 1.2% disall	95.3%	99.7%	95.301	95.93%
**3rsy**	88% core 9.5% allow 1.3% gener 1.3% disall	94.2%	99.2%	92.327	94.01%
**3s4c**	87.5% core 10.0% allow 1.5% gener 1.0% disall	94.4%	99.3%	93.013	94.07%
